# MiR-492 impairs the angiogenic potential of endothelial cells

**DOI:** 10.1111/jcmm.12085

**Published:** 2013-06-26

**Authors:** Francesca Patella, Eleonora Leucci, Monica Evangelista, Brian Parker, Jiayu Wen, Alberto Mercatanti, Milena Rizzo, Elena Chiavacci, Anders H Lund, Giuseppe Rainaldi

**Affiliations:** a Laboratory of Molecular and Gene Therapy Institute of Clinical Physiology, CNRPisa, Italy; b Biotech Research & Innovation Centre, University of CopenhagenCopenhagen N, Denmark; c Department of Biology The Bioinformatics Centre, University of CopenhagenCopenhagen N, Denmark

**Keywords:** microRNA, endothelial cell, eNOS

## Abstract

Endothelial cells growing in high glucose-containing medium show reduced cell proliferation and *in vitro* angiogenesis. Evidence suggests that the molecular pathways leading to these cellular responses are controlled by microRNAs, endogenous post-transcriptional regulators of gene expression. To identify the microRNAs and their targeted genes involved in the glucose responses, we performed the miRNA signature of Human Umbelical Vein Endothelial Cells (HUVECs) exposed and unexposed to high glucose. Among differentially expressed microRNAs, we analysed miR-492 and showed that its overexpression was able to reduce proliferation, migration and tube formation of HUVEC. These effects were accompanied by the down-regulation of eNOS, a key regulator of the endothelial cell function. We showed that eNOS was indirectly down-regulated by miR-492 and we discovered that miR-492 was able to bind mRNAs involved in proliferation, migration, tube formation and regulation of eNOS activity and expression. Moreover, we found that miR-492 decreased VEGF expression in HUVEC and impaired *in vivo* angiogenesis in a tumour xenograft model, suggesting a role also in modulating the secretion of pro-angiogenic factors. Taken together, the data indicate that miR-492 exerts a potent anti-angiogenic activity in endothelial cells and therefore miR-492 seems a promising tool for anti-angiogenic therapy.

## Introduction

Metabolic and physio-chemical stresses have been reported to either promote or inhibit the proliferation and/or the angiogenic properties of endothelial cells. It is known that prolonged growth of endothelial cells in high glucose-containing medium (HG) has an anti-proliferative effect and inhibits angiogenic properties [1–4]. Potential mediators of glucose responses are microRNAs, endogenous 21-25 nucleotides non-coding single-stranded RNA molecules that generally bind to the 3′UTR of target genes and inhibit their translation [2]. Several studies have demonstrated that microRNAs are crucial determinants of endothelial cell behaviour and angiogenesis. We reasoned that a comparative analysis of miRNA signatures of HUVEC either unexposed or exposed to high glucose could allow the identification of numerous microRNAs, which directly or indirectly control genes involved in glucose stress. The challenging issue is the identification of target mRNAs. In fact, each microRNA can potentially bind several (hundreds) of target mRNAs typically through a 6–8 nucleotide complementary region (called microRNA seed), which perfectly pairs with the mRNA 3′UTR sequence. Bioinformatic algorithms (*e.g*. TargetScan, PicTar, Miranda) help to predict putative targets, but the predicted interactions require experimental validation to exclude false positives. So far, several experimental approaches for the identification of microRNA targets have been developed and one of them is the miRNA pull-out technique that allows the identification of all mRNAs targeted by a given microRNA in living cells [5]. In this study, we focused on miR-492, which was one of those differentially expressed in HUVEC grown in HG. We report that the overexpression of miR-492 in HUVEC induced the cellular responses elicited by HG in these cells. Furthermore, we demonstrated that such widespread effects may be due to the ability of miR-492 to bind multiple mRNAs dealing with proliferation and *in vitro* angiogenesis. For these reasons, miR-492 seems likely to become an anti-angiogenic drug.

## Materials and methods

### Reagents

DNA oligos (MWG Biotech, Ebersberg, Germany); double-stranded miR-492 mimic (miR-492), double-stranded negative control (ds-nc) and siRNAs (GenePharma, Shanghai, China); antimiR-492 and scrambled antimiR-492 negative control (sc-nc) (Exiqon, VeVedbaek, Denmark); biotin-tagged thio-uridinylated miR-492 oligos (BioSynthesis Inc., Lewisville, TX, USA); Sepharose high performance beads (GE Healthcare, Cleveland, OH, USA); Matrigel™ Basement Membrane Matrix (BD Bioscience, San Jose, CA, USA); Gene Silencer® (Genlantis, San Diego, CA, USA); Dharmafect 1 (Thermo Scientific, Waltham, MA, USA); Trizol® Reagent, DNAse I amplification grade, Super Script II reverse transcriptase, Taq DNA polymerase, foetal bovine serum (FBS), DMEM medium, Optimem, M199, Lipofectamine2000 (Invitrogen, Carlsbad, CA, USA); miScript Reverse Transcription Kit, MiScript SYBR® Green PCR Kit (Qiagen, Hilden, Germany); TaqMan® MicroRNA Assay (Applied Biosystems, Carlsbad, CA, USA); LightCycler® 480 DNA SYBR Green I Master (Roche, Basel, Switzerland); ALEXA 488M goat antimouse secondary antibody (Molecular Probes, Carlsbad, CA, USA); gelatine, epithelial growth factor (EGF), fraction V bovine serum albumin (BSA), collagenase type II, Crystal violet, D-Glucose, propidium iodide, RNase A, anti-vinculin, VEGF (Sigma-Aldrich, Seelze, Germany); anti-AKT PThr308, anti-caspase 3, anti-PARP, anti-phospho-eNOS (Ser1177), anti-GAPDH (Cell Signaling, Danvers, MA, USA); anti-BRAF, anti-VEGF, anti-SP1 (Santa Cruz, Dallas, TX, USA); anti-eNOS (BD Biosciences); anti-PDPK1 (Upstate, Millipore, Bedford, MA, USA); anti-tubulin (abcam, Cambridge, MA, USA); Cultrex® 96 Well Cell Migration Assay (Trevigen, Gaithersburg, MD, USA); Cell titer 96® AQ_ueous_ One Solution Cell Proliferation Assay, Dual-Luciferase® Reporter Assay System, p-GEM®-T-easy vector, pRL-TK (Promega, Madison, WI, USA); miRCURY™ Array Labelling Kit, miRCURY™ Array microarray slides kit (Exiqon); restriction enzymes (New England BioLabs®, Ipswich, MA, USA).

### Isolation of HUVEC

HUVEC were isolated from human umbilical cords [6]. Briefly, the lumen of an umbilical vein was rinsed with sterile saline and incubated with collagenase II (1 mg/ml) at 37°C for 20 min. Endothelial cells extracted from the vein were grown on gelatine-coated plates in M199 medium supplemented with 10% FBS, EGF (20 ng/ml), heparin (12.5 U/ml), penicillin and streptomycin 1% and L-glutamine 1%. HUVEC between passage 2 and passage 5 were used throughout the experiments.

### Cell transfection

HUVEC (70% confluent) were transfected using either Gene Silencer or Dharmafect 1 transfectant, following the manufacturer's protocol. In the case of Dharmafect 1, medium without antibiotics was used. HUVEC were transfected with either 50 nM siRNAs or 40 nM microRNA, or control ds-nc. HG-HUVEC (cells grown for 72 hrs in medium containing 30 mM glucose) were transfected with antimiR-492 or sc-nc. Cell were collected 48 hrs after transfection and used for all the assays. The transfection efficiency was determined using an oligo FITC (Figure S1).

### Luciferase miRNA target reporter assay

HCT116 Dicer^−/−^ cells were seeded in a 96-well multiplate. The day after, cells were cotransfected with 50 ng pmiR-report 3′UTR target gene +15 ng pRL-TK (Renilla plasmid) and 30 nM miR-492 or miR-191 using Lipofectamine 2000. Cells were assayed 28 hrs after transfection with Dual-Luciferase® Reporter Assay System and luminescence was detected using a luminometer (GloMax-Multi detection system; Promega). The cotransfected pRL-TK was used to normalize the firefly luciferase values of the pmiR-report 3′UTR construct.

### qRT-PCR

Total RNA was extracted from HUVEC using Trizol. To detect microRNAs, 1 μg total RNA was reverse transcribed using miScript Reverse Transcription kit. Forty nanogram of cDNA was used in a 20 μl real time PCR using MiScript SYBR® Green PCR Kit. To detect mRNAs, 1 μg total RNA was reverse transcribed using SuperScriptII reverse transcriptase, upon DNAse treatment. Real time PCR (qRT-PCR) was carried out with LightCycler® 480 DNA SYBR Green I Master (Roche) using LightCycler 480 (Roche).

### miRNA microarray

The miRNA expression profile was performed as previously described [7]. Briefly, total RNA (2 μg) was labelled and manually hybridized to Exiqon miRCURY™ LNA Array 8.0, following the manufacturer's protocol. Differential labelling of total RNA samples with dyes spectrally equivalent to Cy3TM and CyTTM fluorophores allowed comparison of miRNA expression patterns of HG-HUVEC with LG-HUVEC (cells grown in medium containing 5 mM glucose). The labelling method allows selective labelling of miRNA out of the total RNA sample. The hybridized microarrays were scanned using a GenePix 4000B instrument and data were acquired and analysed using GenePix Pro software. Data were normalized with print-tip Loess method by CARMAweb application developed at the Institute for Genomics and Bioinformatics of Graz University of Technology [8].

### Cell proliferation assay

Cell proliferation was checked by seeding 1000 cells on a 96-well plate in triplicate. Cells were assayed with Cell titer 96® AQ_ueous_ One Solution Cell Proliferation Assay at 24 hrs interval. Absorbance at 490 nm was measured after 90 min. using Plate Reader apparatus (SpectraCount, Packard Instrument Company, Downers Grove, IL, USA).

### Tube formation assay

HUVEC were seeded either in a 24-well multiplate (7 × 10^4^/well) or in a 96-well multiplate (0.13 × 10^4^/well) with 2% FBS, on a Matrigel matrix prepared as the manufacturer's recommendation. Crystal violet 4% was added 6 hrs after seeding for 20 min. and finally cells were rinsed in water. Tube formation was evaluated using ImageJ software.

### Migration assay

HUVEC were seeded in M199 0.1% BSA on a Cultrex® 96 Well Cell Migration Assay. 2% FBS was used as chemoattractant. After overnight incubation, cells were assayed according to the manufacturer's protocol, using Calcein-AM as a fluorogenic substrate. Fluorescence was read with a fluorimeter (GloMax-Multi detection system). Number of cell migrated was derived using a standard curve.

### Wound healing assay

VEGF-HUVEC were transfected with either ds-nc or miR-492 and 6 hrs after, a scratch was made with a tip in each dish. Thereafter, one dish was immediately stained with crystal violet and other dishes after 24 hrs to evaluate the wound-healing activity.

### Western blot

Cells were treated with lysis buffer and 25 μg of protein was separated on SDS-PAGE gel and transferred to nitrocellulose membrane. Immunoblots were probed with the primary antibodies. Signals were revealed after incubation with the recommended secondary antibodies coupled to peroxidase using ECL. Scanned images were quantified using Optiquant software.

### Pull-out technique

Pull-out was performed with slight modifications from the original protocol [9]. Briefly, HUVEC were transfected with 1 pmol/l of either miR-492 duplex or a mix of 3′ biotin-tagged miR-492 7tU and miR-492 19 tU duplexes. Cells were washed with PBS and irradiated for 5 min. with long UV (365 nm), 45 μJ/cm^2^, 24 hrs after transfection, using CL-1000 Ultraviolet Crosslinker, UVP, to induce cross-linking of 4-tU nucleotides to RNA. Immediately after this, PBS was removed and TRIzol was added to the plate to extract RNA. After DNAse treatment and RNA quantification, 10 μg RNA was incubated for 2 hrs at 4°C with streptavidin-conjugated beads. After the incubation, two washes in pull-out buffer, followed by two washes in DEPC-treated H_2_O, were performed. The RNA was then recovered by adding TRIzol on the beads and precipitated by NaCl and ethanol. The RNA was amplified and labelled with NuGEN's WT-Ovation FFPE RNA Amplification System. The samples were hybridized to Affymetrix Human Gene 1.0ST expression microarrays.

A differential gene expression analysis was performed between the two data sets: miR-492 duplex and biotin-tagged miR-492 tU (three biological replicates per set). The data were pre-processed by: (*i*) background subtraction; (*ii*) probe summarization (using median polish); (*iii*) median normalization (log_2_ expression − median of each chip's log_2_ expression). The usual quantile normalization was replaced with a simpler, robust median normalization. Differential expression was computed by subtracting biotin-tagged miR-492 tU -492 duplex. Moderated *t*-test (Limma, open source Bioconductor package for R) was used for differential expression analysis across the six biological replicates.

### Statistical analyses

Results are expressed as mean ± SD of at least three independent experiments and data analysed by Student's *t*-test.

## Results

### miR-492 modulates the angiogenic properties of HUVEC

**Fig. 1:**
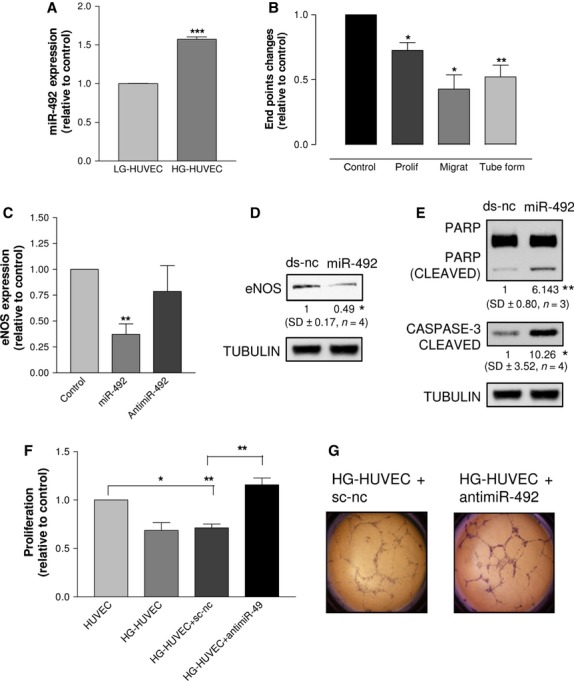
The expression of miR-492 was higher in HG-HUVEC than in LG-HUVEC (**A**). Proliferation, migration and tube formation of HUVEC transfected with miR-492 were significantly reduced in comparison with cells transfected with ds-nc (**B**). HUVEC transfected with either antimiR-492 or miR-492 showed, respectively, unreduced or a reduced expression of eNOS mRNA (**C**). The transfection of miR-492 reduced eNOS protein (**D**) and induced apoptosis as demonstrated by cleavage of caspase 3 and PARP (**E**). HG-HUVEC transfected with antimiR-492 proliferated more than HG-HUVEC transfected with sc-nc (**F**). Representative pictures of tube formation after the transfection of either sc-nc or antimiR-492 in HG-HUVEC are reported in (**G**). Data are reported as mean of at least three independent experiments (*P < 0.05, **P < 0.01, ***P < 0.001, unpaired *t*-test).

**Fig. 2:**
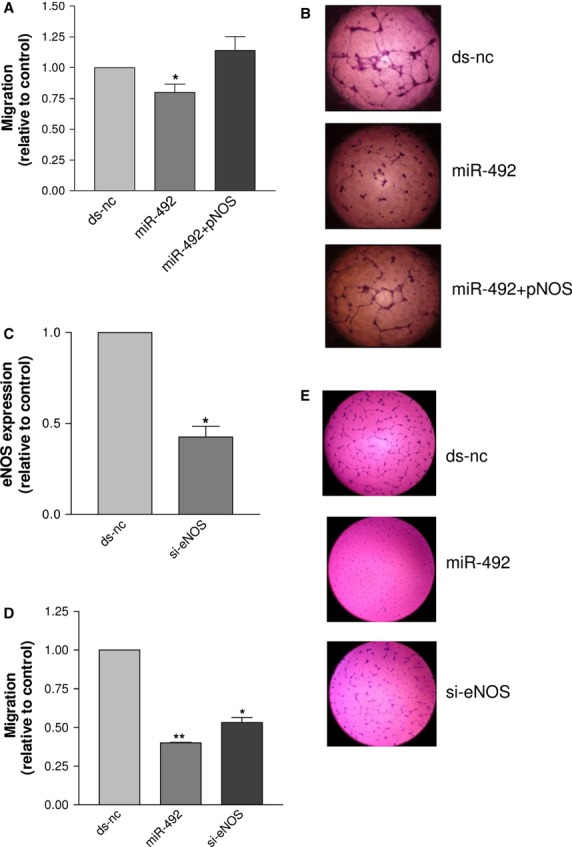
The migration of HUVEC cotransfected with miR-492 and a vector encoding eNOS (pNOS) was higher than that of HUVEC transfected with miR-492 and similar to that of HUVEC transfected with ds-nc (**A**). Representative pictures of tube formation after the transfection of ds-nc (upper), miR-492 (middle) and miR-492+pNOS (bottom) are reported in (**B**), wherein it can be seen that pNOS restored the ability to form tubes. The expression of eNOS (**C**), the migration (**D**) and tube formation (**E**) of HUVEC transfected with either si-eNOS or miR-492 were reduced in comparison to HUVEC transfected with ds-nc. Data are reported as mean of at least three independent experiments (*P < 0.05, **P < 0.01, ***P < 0.001, unpaired *t*-test).

By comparing the miRNA signatures of HG-HUVEC (HUVEC growing in 30 mM glucose-containing medium) and LG-HUVEC (HUVEC growing in 5 mM glucose-containing medium), we found that 14 miRNAs were up-regulated (Table S1). First of all, we validated the expression level of miR-492, which resulted from the microarray data the most up-regulated. The qRT-PCR data showed that miR-492 was 57% more expressed in HG-HUVEC than in LG-HUVEC (Fig. 1A). As the endogenous expression level of miR-492 is lower in HUVEC than in cells of other tissues (Figure S2), it is reasonable that even a small increase like that found in HG-HUVEC might have biological effects. The anti-angiogenic responses elicited by glucose stress consisted in reduced proliferation, tube formation and expression of eNOS (Figure S3). To test whether the overexpression of miR-492 modulates the angiogenic properties of endothelial cells as well as high glucose, we transfected HUVEC with miR-492, and the corresponding control ds-nc. At cellular level, we found that miR-492 consistently inhibited cell proliferation, migration and tube formation (Fig. 1B), down-regulated eNOS at the mRNA level (Fig. 1C) and at the protein level (Fig. 1D). Moreover, miR-492 induced apoptosis as demonstrated by cleavage of caspase 3 and PARP (Fig. 1E). Conversely, the transfection of antimiR-492 was unable to modify the expression of e-NOS (Fig. 1C). To test whether the impairment of angiogenic properties was dependent on the HG-induced overexpression of miR-492, HG-HUVEC were transfected with antimiR-492: HG-HUVEC recovered the ability to proliferate (Fig. 1F) and form tubes (Fig. 1G). On this basis, we concluded that miR-492 impairs the angiogenic properties of HUVEC. To ascertain whether miR-492 exerts the anti-angiogenic activity *in vivo,* we exploited the zebrafish model [10]. We found that xenografts of tumour cells transfected with miR-492 disassembled the vasculature of Tg(kdrl:eGFP)^s843^ zebrafish strain embryo (Figure S4), suggesting that tumour cells transfected with miR-492 have impaired secretion of pro-angiogenic factors. To test the direct involvement of eNOS in determining the anti-angiogenic effects induced by miR-492, HUVEC were cotransfected with miR-492 and a plasmid encoding for eNOS: cell migration (Fig. 2A) and tube formation (Fig. 2B) were rescued by eNOS overexpression, as compared with cells transfected with miR-492. Therefore, we concluded that a relationship between the anti-angiogenic effects of miR-492 and the inhibition of eNOS exists. To verify that the effects of eNOS depletion on HUVEC were similar to those caused by miR-492 overexpression (Fig. 1C), we knocked down eNOS with a short interfering RNA (si-eNOS). We found that si-eNOS was able to reduce eNOS mRNA as much as miR-492 did, reducing it to 50% after 48 hrs from transfection (Fig. 2C). Migration and tube formation of HUVEC were then detected, showing that si-eNOS and miR-492 inhibit these processes to a similar extent (Fig. 2D and E). Thus, it seems that eNOS could mediate the anti-angiogenic effects induced by the overexpression of miR-492.

### eNOS is indirectly controlled by miR-492

**Fig. 3:**
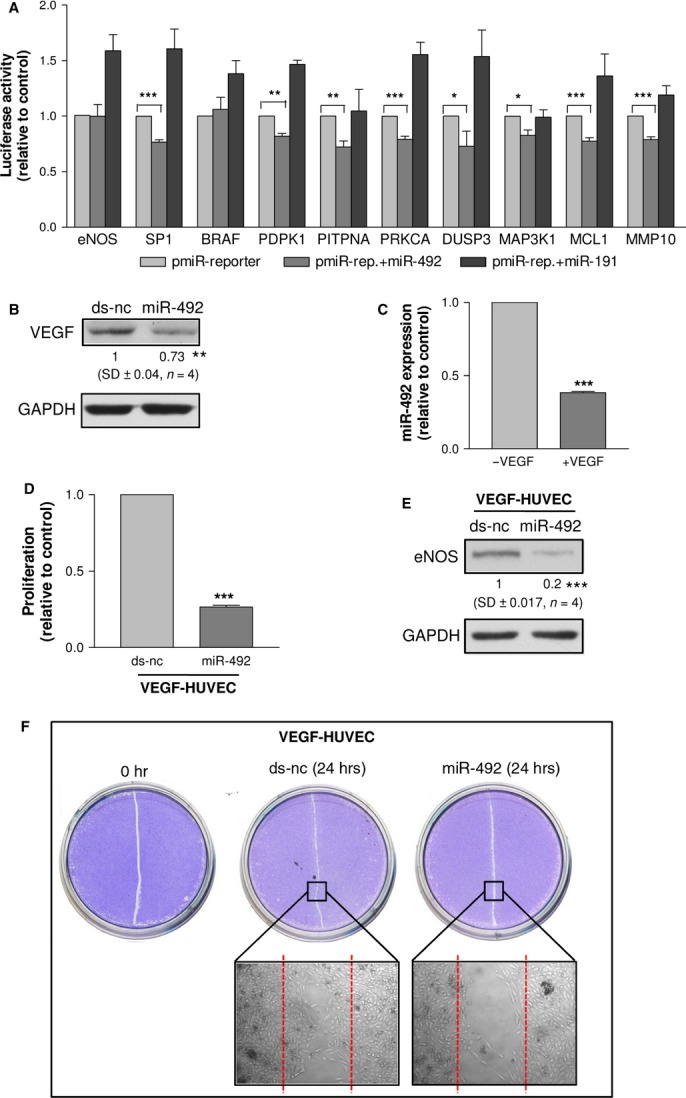
The luciferase assay showed that miR-492 was unable to bind eNOS 3′UTR, whereas the selected miR-492 pull-out targets, except for BRAF, were able to interact with reporters containing putative binding sites in the mRNA target sequence (**A**). HUVEC transfected with miR-492 in comparison to those transfected with ds-nc showed the down-regulation of VEGF (**B**). The expression of miR-492 in VEGF-HUVEC (**C**). VEGF-HUVEC transfected with miR-492 in comparison to those transfected with ds-nc showed the reduction of cell proliferation (**D**), eNOS expression (**E**) and wound-healing activity (**F**). Data are reported as mean of at least three independent experiments (*P < 0.05, **P < 0.01, ***P < 0.001, unpaired *t*-test).

The next issue was to verify whether eNOS was a miR-492 direct target. TargetScan (http://www.targetscan.org) predicted one miR-492 seed match in the 3′UTR of eNOS. To validate the interaction, we cloned the 3′UTR of eNOS mRNA into a luciferase reporter vector. The constructs was cotransfected with either miR-492 or miR-191 [11] as control of the specificity of the binding. As recipient, HCT116 Dicer^−/−^ cells [12] were used, as the binding between mature miRNA/3′UTR is easier detected in cells deficient in mature microRNAs [13]. Unfortunately, we could not detect any decrease in the luciferase activity upon miR-492 overexpression (Fig. 3A). This suggests that eNOS is an indirect target of miR-492 and hence other miR-492 target genes, whose function is to regulate eNOS, should be searched. To unveil which mRNAs are bound by miR-492, we adopted the miRNA pull-out technique. We isolated mRNAs that were bound by either a biotin-tagged miR-492 or a non-biotinylated miR-492 transfected into HUVEC and then compared their mRNA binding profiles. The microarray analysis showed enrichment for the biotin-tagged miR-492, with 268 (75%) of 357 enriched mRNAs, with *P* < 0.01. Only 12.3% (33 of 268) of the enriched mRNAs had canonical 6-mer or stronger seed matches for miR-492 in their 3′UTRs. Looking at mRNAs whose inhibition could explain the inhibition of proliferation and *in vitro* angiogenesis, we identified mRNAs related to apoptosis (MCL1, DUSP), PI3K signalling (PITPNA, PDPK1), proliferation (BRAF, PRKCA, MAP3K1), invasion (MMP10) and eNOS transcription (SP1) (see Table S2 for p-value of the selected genes). Only PITPNA, DUSP3 and PRKCA 3′UTRs show perfect 6-mer seed matches, whereas the other genes have from one (MMP10 and BRAF) to 12 (PDPK1) non-canonical binding sites for miR-492 (retrieved by Miranda, http://www.mirbase.org, see Table S3 for binding site details).

To rule out the possibility that the nine selected pull-out genes were bound in a non-specific manner by miR-492, their 3′ UTRs were cloned into pmiR-reporter vector. The luciferase assay showed the interaction of miR-492 with the cloned mRNA 3′UTRs (with the exception of BRAF), despite the presence of non-canonical binding sites for miR-492 (Fig. 3A). Nevertheless, western blot verified BRAF down-regulation by miR-492 (Figure S6). To conclude, we identified eight direct miR-492 targets that mediate the effects on proliferation, migration, tube formation and eNOS regulation.

In the angiogenesis process, VEGF is important, but the miRNA pull-out assay revealed that miR-492 was unable to bind VEGF mRNA. To check for an indirect control, HUVEC were transfected with either miR-492 or its relative control (ds-nc). We found that miR-492 reduced the expression of VEGF (Fig. 3B). Then, we asked whether HUVEC once stimulated with VEGF (named VEGF-HUVEC) could change the sensitivity to miR-492. After setting up the experimental conditions to obtain VEGF-HUVEC (Figure S6), we measured miR-492 in VEGF-HUVEC. Remarkably, we found that VEGF induced a decrease in miR-492 expression (Fig. 3C). We then transfected VEGF-HUVEC with either ds-nc or miR-492. After 48 hrs from transfection, miR-492 strongly reduced the proliferation of VEGF-HUVEC (Fig. 3D) and the expression of e-NOS (Fig. 3E). The wound healing assay confirmed that VEGF-HUVEC transfected with miR-492 were unable to heal the wound in comparison with VEGF-HUVEC transfected with ds-nc (Fig. 3F).

### SP1 and PDPK1 link miR-492 expression to eNOS regulation

**Fig. 4:**
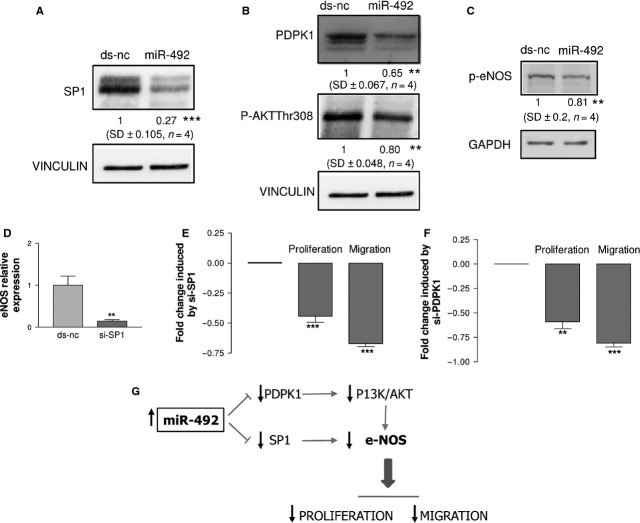
HUVEC transfected with miR-492 showed the down-regulation of SP1 (**A**), PDPK1 and PI3K/AKT (**B**) and p-eNOS (**C**). HUVEC transfected with si-SP1 showed a reduction in eNOS expression (**D**), proliferation and migration (**E**). HUVEC transfected with si-PDPK1 showed the reduction in proliferation and migration (**F**). Schematic representation of miR-492 network (**G**). Data are reported as mean of at least three independent experiments (*P < 0.05, **P < 0.01, ***P < 0.001, unpaired *t*-test).

SP1 and PDPK1, two of the validated miR-492 direct targets (Fig. 3A), are known, respectively, as transcriptional regulator [14] and activator [15, 16] of eNOS. Thus, we expected that both proteins should be down-regulated in miR-492-transfected HUVEC. Indeed, SP1 (Fig. 4A) and PDPK1 (Fig. 4B) proteins were down-regulated. Moreover, consequent to the down-regulation of PDPK1 (Fig. 4B), its downstream target P-Akt Thr308 (Fig. 4B) as well as its target p-eNOS (Fig. 4C) was accordingly down-regulated in miR-492-transfected HUVEC. To prove that either SP1 or PDPK1 can modulate the anti-angiogenic properties of HUVEC, we silenced these genes. We observed that si-SP1 decreased the eNOS transcription (Fig. 4D), proliferation and migration (Fig. 4E) confirming other results [17] and inhibited tube formation (Fig. 4G). Similarly, the PDPK1 silencing decreased the proliferation and migration of HUVEC (Fig. 4F) and inhibited tube formation (Fig. 4G). The scenario depicted in HUVEC sees miR-492 negatively regulating both SP1, which controls the transcription of eNOS, and PDPK1, which controls eNOS activation *via* the inhibition of PI3K/AKT pathway. In case of up-regulation of miR-492, eNOS expression is reduced and concomitantly proliferation and *in vitro* angiogenesis are impaired (Fig. 4H). However, as miR-492 regulates other targets involved in proliferation and *in vitro* angiogenesis, we can speculate that proliferation and migration are in part inhibited *via* PDPK1 and SP1.

## Discussion

High glucose-containing medium is an anti-proliferative stimulus and reduces the ability of endothelial cells to migrate and form tubes. That prompted us to ask whether microRNAs might be mediators of the HG-induced phenotypes. By comparing the miRNA signatures of HG-HUVEC and LG-HUVEC, we discovered that miR-492 was up-regulated in HG-HUVEC. It is of note that among the microRNA up-regulated by HG, we also got miR-125a and miR-320, which were previously found to be overexpressed in type 2 diabetes Goto Kakizaki rats, where they were induced in insulin-targeted tissues (liver and adipose tissue) and in microvascular endothelial cells (MMVEC) respectively [18, 19]. Conversely, other microRNAs, despite being reported to be involved in high glucose-induced responses, were not considered as their fold change felt under our applied threshold. This was the case of miR-503, which has a prominent role in impairment of post-ischemic reparative angiogenesis in the setting of diabetes [20] and of miR-221, which has been showed to be induced by high levels of glucose in HUVEC [21] and caused vascular damage in mouse model [22].

Little information is available in the literature on the role of miR-492. It is only known that its expression levels are very low in every tissues in normal conditions [23] and that it is conserved only in primates and horses [24]. In cultured cells, miR-492 is up-regulated by the tumour suppressor p53 [25]. Moreover, a microarray profiling of stage II colon cancers revealed that miR-492 was down-regulated in cancers with recurrence of metastases in the liver and/or lungs [26]. Thus, it seems that the expression of miR-492 inversely correlates with metastases formation, an observation that supports an anti-angiogenic role for miR-492. We discovered that miR-492 is involved in *in vitro* angiogenesis as it was able, when overexpressed, to mimic the glucose stress and, when depleted from HG-treated cells, to restore the ability of HUVEC to proliferate and form tubes. An additional finding was that miR-492 regulates and is regulated by VEGF and that the overexpression of miR-492 was able to abolish the angiogenic potential of VEGF. Moreover, we discovered that miR-492 inhibited *in vivo* angiogenesis promoted by tumour cells engrafted in a zebrafish model. These findings make miR-492 a promising tool to be tested in tumours as anti-angiogenic drug and suggest a role of miR-492 in modulating the secretion of pro-angiogenic factors.

One of the molecular targets of miR-492 was eNOS. We showed that the transfection of miR-492 decreased eNOS expression and that the silencing of eNOS led to similar anti-angiogenic phenotypes as miR-492 overexpression. The presence of a functional link between miR-492 and eNOS was further supported by the findings that the overexpression of eNOS together with miR-492 rescued the ability of HUVEC to migrate and form tubes. It is of note that, despite a binding site for miR-492 in eNOS 3′UTR was predicted by a bioinformatic algorithm, both the luciferase and the pull-out assays were unable to detect a positive miR-492/3′UTR eNOS interaction. The conclusion is that eNOS is an indirect target of miR-492. Efforts in understanding how miR-492 might regulate eNOS were then made. The selected technique was the pull-out, which allows the identification of mRNAs bound by miR-492 in living HUVEC. Several important novel findings came out. Firstly, the list of identified mRNAs was deeply different from the list of genes predicted by the most common informatic tools. Secondly, among the apoptosis (MCL1, DUSP), PI3K signalling (PITPNA, PDPK1), proliferation (BRAF, PRKCA, MAP3K1), invasion (MMP10) and eNOS transcription (SP1)-related genes, the percentage of those with a canonical binding site (6-mer seed match) for miR-492 was lower than expected, nevertheless eight of nine of selected mRNAs were positive at luciferase assay. We could not validate the binding of miR-492 to BRAF 3′UTR, but as we proved that BRAF was regulated by miR-492 at protein level, it is likely that the actual binding site for BRAF does not reside in the portion of 3′UTR we cloned, but somewhere else in the 5′UTR or in the coding region.

The presence of non-canonical miRNA binding sites able to regulate expression has just recently emerged as a widespread phenomenon [27]. These findings indicate that the pull-out technique identifies true miRNA/mRNA interactions and also suggest that positive interactions can occur in the absence of a canonical-binding site.

In addition, our results highlighted the importance of sperimentally identifying microRNA targets, especially for the low expressed microRNAs, as miR-492, because they tend to have recent origin (*e.g*. primates), thus conservation cannot be invoked as a filter to remove false sites, as used by some bioinformatic algorithms [28].

Finally, we propose a network wherein miR-492 negatively regulates eNOS and the angiogenic properties of endothelial cells through the down-regulation of SP1 and PDPK, two validated targets of miR-492, which act, respectively, as transcriptional regulator of eNOS and activator of eNOS, probably *via* the inhibition of PI3K/AKT pathway.

In conclusion, we showed that microRNAs are mediators of cellular responses induced by glucose stress and that miR-492 has a strong anti-angiogenic action in HUVEC because of its ability to bind mRNAs involved in proliferation, migration, tube formation and to inhibit indirectly the expression and activity of eNOS. This suggests that the anti-angiogenic phenotype is due to the concomitant impairments of several gene expression networks, whose characterization is of fundamental importance in the prospective of using miR-492 as anti-angiogenic or its inhibitor as a pro-angiogenic RNA-based drug.

## Supporting information

**Table S1.** microRNAs differentially expressed (high glucose versus normal glucose).

**Table S2.** p- value of miR-492 pull-out genes.

**Table S3.** Binding sites proposed by Miranda between miR-492 and pull-out targets.

**Figure S1.** Transfection efficiency of HUVEC.

**Figure S2.** Expression of miR-492 in human tissues.

**Figure S3.** Effects of high glucose concentration (30 mM) on HUVECs.

**Figure S4.** Zebrafish/tumor xenograft angiogenesis assay.

**Figure S5.** BRAF protein expression after miR-492 transfection.

**Figure S6.** Set up and transfection of VEGF-HUVEC.

**Data S1.** Supplemental references.
