# Supplementary material for: MiR-492 impairs the angiogenic potential of endothelial cells
Source: J Cell Mol Med. 2013 Jun 26;17(8):1006–15. doi: 10.1111/jcmm.12085 (PMC3780533; doi:10.1111/jcmm.12085)
Supplement: Supplementary file 1 [file jcmm0017-1006-SD1.pdf]

SUPPLEMENTAL MATERIAL

TABLES

Table S1.

microRNAs differentially expressed (high glucose versus normal glucose). Total RNA extracted from HG-HUVEC and LG-HUVEC-LG was used for detection of miRNA differentially expressed as already described [1].

Briefly, total RNA (2 μg) was labelled and manually hybridized to Exiqon miRCURY^{TM} LNA Array 8.0, following the manufacturer's protocol. Differential labelling of total RNA samples with dyes spectrally equivalent to Cy3TM and CyTTM fluorophores allowed comparison of miRNA expression patterns of HG-HUVECs and LG-HUVECs. The labelling method allows selective labelling of miRNAs out of the total RNA sample. The hybridised microarrays were scanned using a GenePix 4000B instrument and data were acquired and analysed using GenePix Pro software. Data were normalized with print-tip Loess method by CARMAweb application developed at Institute for Genomics and Bioinformatics of Graz University of Technology [2].

microRNA	*Z score*
hsa-miR-325	-2,0666
hsa-miR-184	2,0596
hsa-miR-493-5p	2,2321
hsa-miR-542-3p	2,2736
hsa-miR-452	2,4130
hsa-miR-335	2,4929

hsa-miR-487b	2,6023
hsa-miR-320	2,6462
hsa-miR-375	2,6580
hsa-miR-125a-5p	2,6923
hsa-miR-373*	2,9835
hsa-miR-494	3,0677
hsa-miR-299-3p	3,2511
hsa-miR-492	4,9014

Table S2. p- value of miR-492 pull-out genes.

Gene name	p- value	Reference
PDPK1	4.6465e-05	[3]
DUSP	0.000250645	[4]
BRAF	0.000354541	[5]
PITPNA	0.000420569	[6]
MMP10	0.000520498	[7]
SP1	0.00069935	[8]
MAP3K1	0.00679158	[9]
MCL1	0.00766584	[10]
PRKCA	0.00875125	[11]

Table S3. Binding sites proposed by Miranda between miR-492 and pull-out targets

```
Gene name gene_start gene_end align_len miRNA_3_5 alignment          ID %    Similar %   lenght 3'UTR

MMP10        80     101     19    uucUUAGAACAGGGCGUCCAGGa          73.68     89.47       290
                                     |||:|| |||:||| ||:|
                                  gttAATTTT-TCCTGCATGTTCt

PDPK1      1375    1404     27    uuCUUA----GA--ACAGGG-CGUCCAGGa   59.26     70.37       5421
                                    ||:|    ||  || ||: |||||||:
                                  gaGAGTGCTCCTGGTGGCCTGGCAGGTCTg

PDPK1      3700    3722     20    uuCUUAGAACAGGGCGUCCAGGa          75        85          5421
                                    ||||||||| |: |: ||||
                                  caGAATCTTGTACTTCGTGTCCa

PDPK1      3935    3957     11    uucuuagaacaGGGCGUCCAGGa          72.73     90.91       5421
                                             ||: ||||||:
                                  gtggggccccaCCTTCAGGTCTt

PDPK1      1668    1690     14    uucuuagaACAGGGCGUCCAGGa          78.57     85.71       5421
                                          |||:|| |||| ||
                                  ggcagagaTGTTCCCCAGGCCCt

PDPK1       624     646     18    uucuUAGAACAGGGCGUCCAGGa          61.11     77.78       5421
                                      :| ||| |  ||||||::
                                  tgctGTGTTGGCAGGCAGGTTTg

PDPK1      1499    1526     24    uucUUAGAACAGG-----GCGUCCAGGa     58.33     62.5        5421
                                     || : |||||     | | |||||
                                  ctcAACTGTGTCCACCCTCCCTGGTCCc

PDPK1      1554    1580     24    uuCUUAGA-ACAGGG--C-GUCCAGGa      66.67     66.67       5421
                                    ||  || ||| ||  | ||| |||
                                  gtGACCCTGTGTGCCAGGCCAGATCCa

PDPK1      2622    2642     14    uucuuagaACAGGGCGUCCAGGa          78.57     78.57       5421
                                          |||||  ||||| |
                                  ggcctaggTGTCC--CAGGTGCc

PDPK1      1920    1942     23    uuCUUAGA-ACAGGGCGUCC--AGGa       65.22     73.91       5421
                                    ||||:| |||:   ||||  |||
                                  agGAATTTCTGTT---CAGGCTTCCt

PDPK1       375     394     19    uucUUAGAACAGGGCGUCCAGGa          63.16     68.42       5421
                                     || : |||   || |||||
                                  cccAACTGTGT---GCTGGTCCt

PDPK1      4069    4095     24    uuCUUA-GAACAG---GGCGUCCAGGa      62.5      62.5        5421
                                    || | | || |   | ||| ||||
                                  caGATTGCCTGGCTGGCAGCAAGTCCa

PDPK1      3467    3489     19    uucUUAGAACAGGGCGUCCAGGa          57.89     84.21       5421
                                     |:|: ||||:: ||| |:|
                                  cttAGTTGTGTCTTACAGATTCt

BRAF        316     346     27    uucUUAGAACA--GG------GCGUCCAGGa  55.56     59.26       584
                                     | |:||||  ||      | || ||||
                                  gcaACTTTTGTTGCCAGCTATCACATGTCCa

MAP3K1     1944    1964     20    uuCUUAGAACAGGGCGUCCAGGa          70        85          2471
                                    ||:| |||| ::|||| |||
                                  ttGAGT-TTGT-TTGCAGTTCCc

MAP3K1      824     846     18    uucUUAGAACAGGGCGUCCAGga          66.67     83.33       2471
                                     | |||| |: :||||||:
                                  accACTCTTATTGTGCAGGTTaa

MAP3K1      442     465     21    uuCUUAGAAC--AGGGCGUCCAGga        61.9      76.19       2471
                                    | ||| ||  ||:: :|||||
                                  caGTATC-TGCCTCTTTTAGGTCag

PITPNA      907     931     19    uucuuAGA-AC-AGGGCGUCCAGGa        68.42     84.21       2572
                                       ||| || |::| :||||||
                                  ttttcTCTGTGCTTTCTTAGGTCCc

PITPNA      524     546     19    uucUUAGAACAGGGCGUCCAGGa          63.16     84.21       2572
                                     ||| | ||:::|||| ||:
                                  agcAATGTGGTTTTGCAGCTCTg

PITPNA       44      63     21    uuCUUAGAACAGGG-CGUCCAGGa         66.67     66.67       2572
                                    |||    | ||| |||| |||
                                  agGAA----GACCCAGCAGCTCCa

PITPNA     1559    1581      6    uucuuagaacagggcgUCCAGGa          100       100         2572
                                                  ||||||
                                  gcgctgaccttcattgAGGTCCc

PITPNA     2316    2338      6    uucuuagaacagggcgUCCAGGa          100       100         2572
                                                  ||||||
                                  gccttctcatgcagagAGGTCCg
```

Table S3 (continue).

SP1	1093	1117	24	uuCUUAGAACAGGGC----GUCCAGGa ||| || |:|| |||||:| agGAA--ATGACTCGTAGTCAGGTTCa	58.33	66.67	5202
SP1	4583	4610	25	uuCUUAGAACA---G--GGCGUCCAGGa ||| ||||	|||||||:<br>ttGAACATTGTGGACAAGGGCAGGTCTt	60	64	5202
SP1	1275	1294	20	uuCUUAGAACAGGGCGUCCAGGa ||||| |||:: |||||::<br>gtGAATC-TGTTT--CAGGTTTc	65	85	5202
SP1	3650	3672	20	uuCUUAGAACAGGGCGUCCAGGa ||:|:||| ||||| | aaGAGTTTTGGAGATCAGGTACc	60	70	5202
SP1	789	807	19	uucUUAGAACAGGGCGUCCAGGa |||:||	|||| || gtcAATTTTTT----CAGGACCt	63.16	68.42	5202
SP1	1217	1245	23	uucuuAGAAC----AGGG--CGUCCAGGa |:||| |||| :||||:| acttcTTTTGAACATCCCCACTAGGTTCt	56.52	69.57	5202
SP1	1908	1933	22	uucUUAG---AACAGGGCGUCCAGGa<br>:||| || |:: |||| || tcaGATCAAAATGCCTTTCAGGCCCa	54.55	68.18	5202
MCL1	1343	1369	20	uucuuaGAACA----GGGCGUCCAGGa |||||		||||||:<br>ggcttgCTTGTTACACACACAGGTCTa	65	70	2816
MCL1	1692	1718	23	uuCUUAGAAC-AGGG----CGUCCAgga ||| |||| |||: |||||| acGAA-CTTGATCCTGTTAGCAGGTggt	69.57	73.91	2816
MCL1	903	928	22	uucUUAGA--ACAGG-GCGUCCAGGa |||||	|: :||||||::<br>acaAATCTGATAACTATGCAGGTTTa	59.09	77.27	2816
MCL1	824	848	21	uucUUAGAACAGGGC--GUCCAGGa || :||||:|:| ||| ||:<br>ctaAACTTTGTTCTGTTCAGTTCTa	61.9	80.95	2816
DUSP3	10	32	6	uucuuagaacagggcgUCCAGGa |||||| accaccucugcucgagAGGUCCg	100	100	3505
DUSP3	330	352	6	uucuuagaacagggcgUCCAGGa |||||| ccuaagaugcucacagAGGUCCc	100	100	3505
DUSP3	509	531	6	uucuuagaacagggcgUCCAGGa |||||| gcccucucacgucuugAGGUCCg	100	100	3505
DUSP3	1034	1056	6	uucuuagaacagggcgUCCAGGa |||||| caagcagggaagugaaAGGUCCc	100	100	3505
DUSP3	1375	1097	6	uucuuagaacagggcgUCCAGGa |||||| acucugggcaaagagaAGGUCCu	100	100	3505
DUSP3	2888	3010	6	uucuuagaacagggcgUCCAGGa |||||| aucuagaagaccucuaAGGUCCc	100	100	3505
PRKCA	2047	2069	6	uucuuagaacagggcgUCCAGGa |||||| gagacacccccaugcaAGGUCCu	100	100	6724
PRKCA	3085	3107	6	uucuuagaacagggcgUCCAGGa |||||| gccgcagccacgcaggAGGUCCc	100	100	6724

SUPPLEMENTAL MATERIAL

FIGURES

Figure S1.

Transfection efficiency of HUVEC. Exponentially growing HUVECs were transfected with 80nM oligo-FITC using Gene Silencer transfectant. After 24 hours HUVECs were collected and analyzed with BD-FACscalibur. FACS analysis showed that approximately 98% of cells were FITC-positive (Figure S3) and therefore we can reasonably assume that the various RNA molecules under testing were always efficiently transfected.

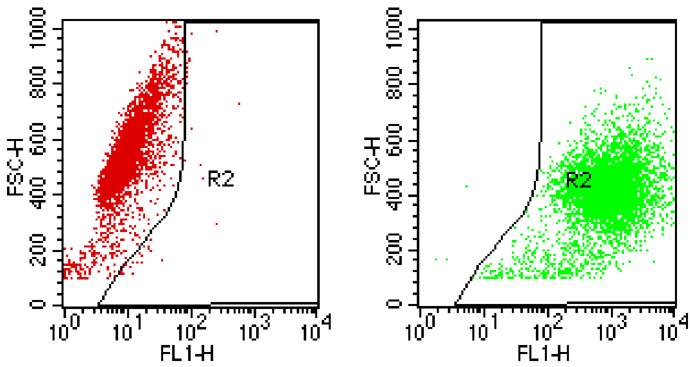

Figure S1. Scattergram analysis of HUVEC untransfected (left panel) and transfected (right panel) with oligo FITC. Transfection efficiency was ~98%.

Figure S2.

Expression of miR-492 in human tissues. Total RNA was extracted from $1x10^6$ HUVEC cells using miRNAeasy mini kit (Qiagen) following the manufacturer's recommendation. Total RNA from prostate, colon and lung tissue were purchased from Ambion (FirstChoise Human Total RNA). Total RNA were reverse transcribed using miScript Reverse Transcription kit (Qiagen) and miR-492 were quantified by qRT-PCR carried out with Rotor-Gene Q 2-Plex using QuantiTect SYBER Green PCR kit (Qiagen).

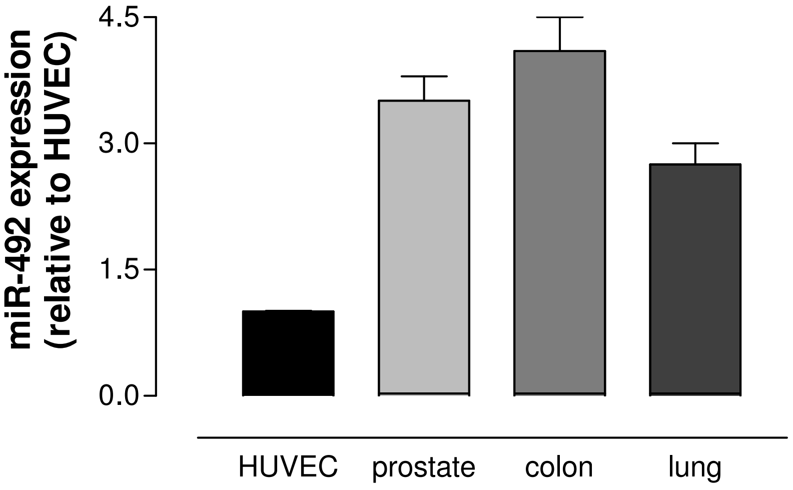

Figure S2. miR-492 expression level in HUVEC and human tissues

Figure S3.

Effects of high glucose concentration (30 mM) on HUVECs. We cultivated HUVEC in either high glucose-(HG-HUVEC, 30mM) or low glucose-(LG-HUVEC, 5mM) containing medium for 72 hours. HG-HUVEC were less proliferating than LG-HUVEC (Figure S3A), showed reduced ability to form tubes (Figure S3B) and down-regulation of eNOS, a key regulator of angiogenesis (Figure S3C).

A

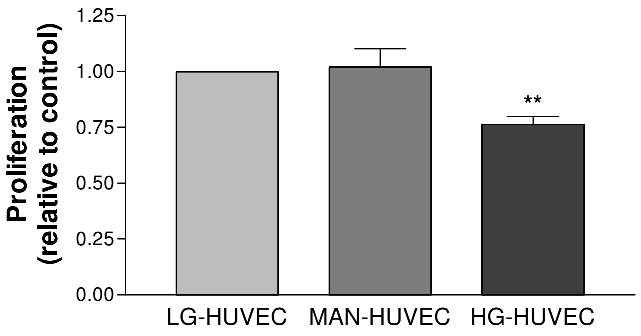

B

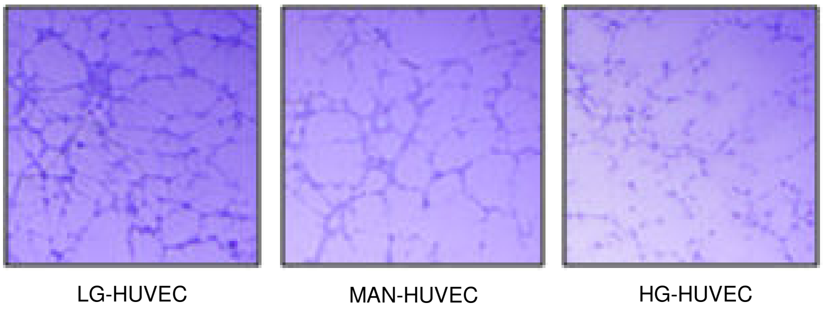

C

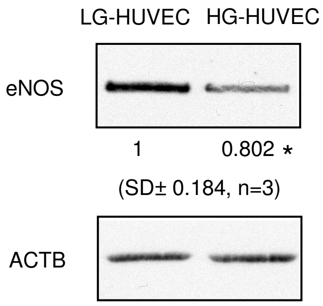

Figure S3. (A) Relative proliferation measured by MTS assay; **(B)** tube formation on matrigel; **(C)** eNOS protein expression. Mannitol 30 mM (MAN-HUVEC) was used as osmotic pressure control.

Figure S4.

Zebrafish/tumor xenograft angiogenesis assay. The zebrafish/tumor xenograft angiogenesis assay was performed as described by Nicoli and Presta [12]. DU-145 prostate cancer cells were transfected with 80 nM double stranded miR-492 mimic (miR-492) or negative control (ds-nc) using Polyfect Transfection Reagent (Qiagen) according to the manufacturer's instruction. After 48 hours cells were collected, and suspended in Cultrex BME (Basement Membrane Extract (R&D System). Tg(kdrl:eGFP)^{s843} zebrafish were raised in standard laboratory conditions (Westerfileld M zebrafish book) in a ZEBTEC Zebrafish Housing System (Tecniplast, Varese, Italy). Embryos were injected 48h post-fertilization and analyzed 24h post injection. For imaging embryos were treated with 0,01mM tricaine solution (Sigma-Aldric) and embedded in 1,2% low melting agarose medium. Stacks were acquired with a Leica TCS-SL DM IRE 2 confocal microscope and image stacks were processed with FIJI-WIN32 by projection.

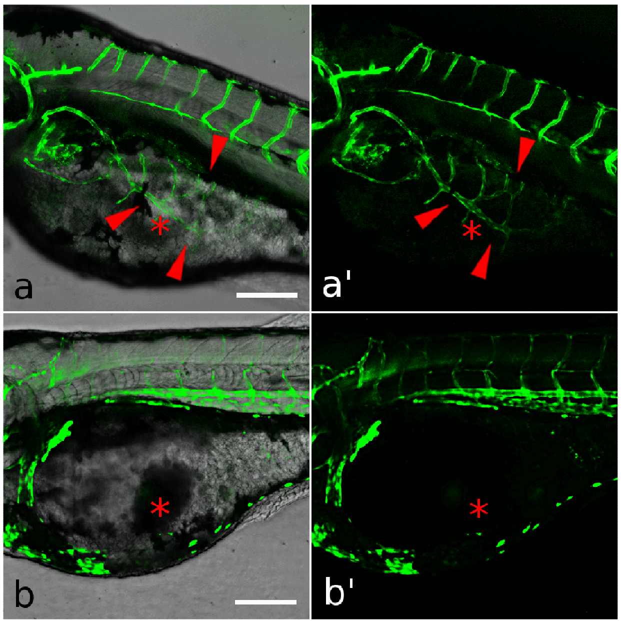

Figure S4. SX: zebrafish/tumor xenograft angiogenesis assay performed in Tg(kdrl:eGFP)^{s843} zebrafish strain: **(a)**, **(a')** 72hpf embryos injected with DU-145 cells transfected with ds-nc show new vessels (n= 22/22 embryos). **(b)**, **(b')** 72hpf embryos injected with DU-145 cells transfected with miR-492 show no angiogenetic response (n=17/20 embryos). Red star: site of injection, red arrows: new vessels, white scalebar: 150 µm.

Figure S5.

BRAF protein expression after miR-492 transfection. The luciferase assay ruled out the interaction of miR-492 with the cloned mRNA 3'UTR BRAF. Nevertheless, western blot verified BRAF down-regulation by miR-492.

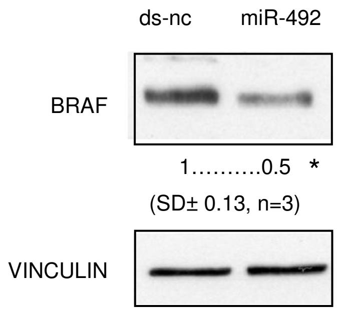

Figure S5. BRAF protein expression level in HUVEC transfected with either miR-492 or ds-nc.

Figure S6.

Set up and transfection of VEGF-HUVEC

Exponentially growing HUVEC p5 were seeded at cell density of $2x10^4/cm^2$ in EBM2 (Lonza) plus modified bullet kit (without VEGF) and containing 0.4% FBS. After 24 hours different concentrations of VEGF were added and 24 hours later the proliferation was measured as following. 7.5 10^3 cells per 24 well multiplate were seeded and at specified time points cells were fixed in 2% paraformaldehyde in PBS and subsequently stained with 0.1% crystal violet dissolved in 20% methanol and let dry at room temperature. Then, cells were lysed with acetic acid 10% and the optical density (OD 590 nm) of the solution, detected with Plate Reader apparatus (SpectraCount, Packard), was used to measure cell proliferation.

From dose-response curve (Figure S6A) we selected 10ng/ml VEGF and after 24 hours exposure to VEGF we measured expression level of miR-492, eNOS and its phosphorylated form by Western blot and found that miR-492 was downregulated (Figure S6B) whereas eNOS and p-eNOS were upregulated (Figure S6C and S6D). Finally we tested the susceptibility of VEGF-HUVEC to be transfected by using an oligoFITC. The FACScan analysis showed that approximately 70% of VEGF-HUVEC were fluorescent thus indicating that VEGF-HUVEC retained the ability to be transfected (Figure S6E).

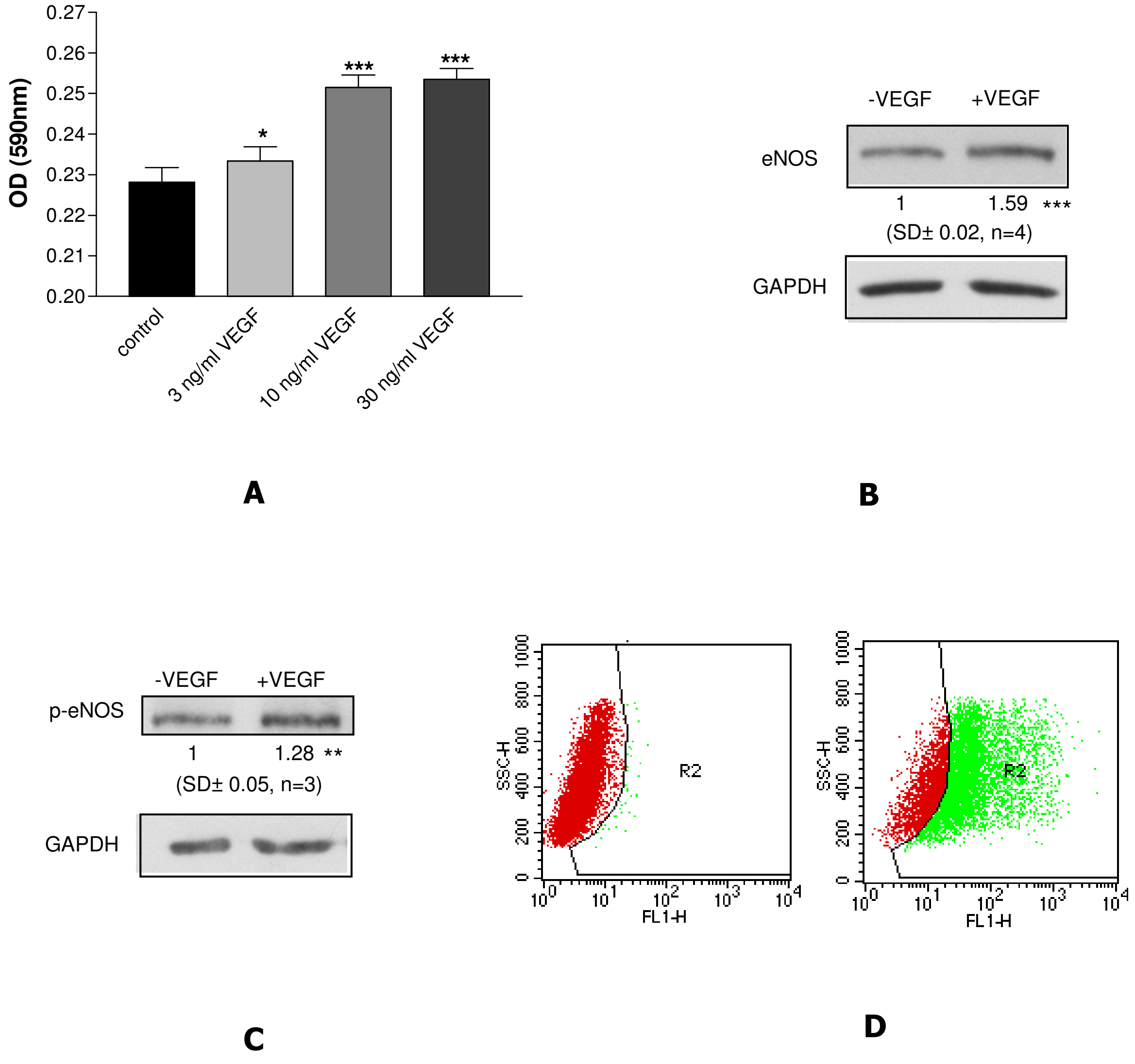

Figure S6. (A) VEGF dose-response curve; eNOS **(B)** and p-eNOS **(C)** quantification; **(E)** Scattergram analysis of VEGF-HUVEC untransfected (left panel) and transfected (right panel) with oligo FITC. Transfection efficiency was ~70%.

SUPPLEMENTAL REFERENCES

1. **Pitto L, Rizzo M, Simili M, et al.** miR-290 acts as a physiological effector of senescence in mouse embryo fibroblasts. *Physiological genomics*. 2009 Nov 6;39(3):210-8.
2. **Rainer J, Sanchez-Cabo F, Stocker G, Sturn A, Trajanoski Z.** CARMAweb: comprehensive R- and bioconductor-based web service for microarray data analysis. *Nucleic Acids Res*. 2006 Jul 1;34(Web Server issue):W498-503.
3. **Wilhelm SM, Carter C, Tang L, et al.** BAY 43-9006 exhibits broad spectrum oral antitumor activity and targets the RAF/MEK/ERK pathway and receptor tyrosine kinases involved in tumor progression and angiogenesis. *Cancer Res*. 2004 Oct 1;64(19):7099-109.
4. **Heo SH, Choi YJ, Ryoo HM, Cho JY.** Expression profiling of ETS and MMP factors in VEGF-activated endothelial cells: role of MMP-10 in VEGF-induced angiogenesis. *J Cell Physiol*. Sep;224(3):734-42.
5. **Townsend KJ, Trusty JL, Traupman MA, Eastman A, Craig RW.** Expression of the antiapoptotic MCL1 gene product is regulated by a mitogen activated protein kinase-mediated pathway triggered through microtubule disruption and protein kinase C. *Oncogene*. 1998 Sep 10;17(10):1223-34.
6. **Wojnowski L, Zimmer AM, Beck TW, et al.** Endothelial apoptosis in Braf-deficient mice. *Nat Genet*. 1997 Jul;16(3):293-7.
7. **Arnoldussen YJ, Saatcioglu F.** Dual specificity phosphatases in prostate cancer. *Mol Cell Endocrinol*. 2009 Oct 15;309(1-2):1-7.
8. **Zhang R, Min W, Sessa WC.** Functional analysis of the human endothelial nitric oxide synthase promoter. Sp1 and GATA factors are necessary for basal transcription in endothelial cells. *The Journal of biological chemistry.* 1995 Jun 23;270(25):15320-6.
9. **Primo L, di Blasio L, Roca C, et al.** Essential role of PDK1 in regulating endothelial cell migration. *J Cell Biol*. 2007 Mar 26;176(7):1035-47.
10. **Partovian C, Simons M.** Regulation of protein kinase B/Akt activity and Ser473 phosphorylation by protein kinase Calpha in endothelial cells. *Cellular signalling.* 2004 Aug;16(8):951-7
11. **Kauffmann-Zeh A, Thomas GM, Ball A, et al.** Requirement for phosphatidylinositol transfer protein in epidermal growth factor signaling. *Science.* 1995 May 26;268(5214):1188-90.
12. **Nicoli S, Presta M**.The zebrafish/tumor xenograft angiogenesis assay. *Nature Protocols* 2007 (2): 2918 - 2923.
